# How Changing Portraits and Opinions of “Pit Bulls” Undermined Breed-Specific Legislation in the United States

**DOI:** 10.3390/ani15142083

**Published:** 2025-07-15

**Authors:** Michael Tesler, Mary McThomas

**Affiliations:** Department of Political Science, University of California, Irvine, CA 92697, USA

**Keywords:** pit bulls, public opinion, public policy, breed-specific legislation, policy narratives, social construction of target populations

## Abstract

Scholars and journalists typically trace the diffusion of breed-specific legislation (BSL) in the U.S. to the surge in negative media portraits of pit bull-type dogs (PBTDs) during the late twentieth century. Yet, while news coverage still portrays these dogs unfavorably, we document a sharp rise in countervailing sources of “pit bull positivity” over the past two decades. Drawing on insights from the respective social science research on changes in attitudes and public policy, we argue that this influx of positivity should powerfully impact opinions and policies towards PBTDs. Our data and analyses consistently support that argument. We analyze two different series of repeated cross-sectional surveys to show that public support for “pit bulls” grew considerably from 2014 to 2024. We also show that voters’ support for ballot measures overturning local “pit bull bans” increased substantially during that same ten-year period. Finally, our analysis of the frames and narratives deployed in recent state and local policy debates shows how this growing pit bull positivity has helped overturn over 300 discriminatory laws against these dogs since 2012. We conclude with a discussion of how shifts in portraits and opinions of PBTDs will likely continue eroding breed-specific legislation going forward.

## Simple Summary

This article explains how the rise in positive portraits of pit bull-type dogs on social media and in popular culture helped change public opinion and policies. Drawing on insights from the respective social science research on changes in attitudes and public policy, we argue that this influx of positivity should powerfully impact opinions and policies towards pit bull-type dogs. In keeping with that contention, our analyses of nationally representative survey data show that public support for “pit bulls” grew considerably from 2014 to 2024. We also show that voters’ support for ballot measures overturning local “pit bull bans” increased substantially during that same ten-year period. Finally, we analyze recent state and local policy debates to show how this growing “pit bull positivity” has helped overturn over 300 discriminatory laws against these dogs since 2012.

## 1. Introduction

Pit bull-type dogs (PBTDs), such as the American Pit Bull Terrier, the Staffordshire Bull Terrier, and the American Staffordshire Terrier, have long faced widespread prejudice and discrimination in the United States. A growing body of scientific research shows that these dogs’ temperaments/behaviors are comparable with other breeds and that breed is generally a poor predictor of a dog’s individual behavior) [1,2,3,4,5,6,7,8,9]. Nevertheless, surveys show that a large share of the American public negatively stereotypes “pit bulls” as aggressive/scary and rates them significantly less favorably than other well-known breeds [10,11]. Those prevalent beliefs are implicated in PBTDs’ excessively high kill rates in animal shelters where they accounted for over 40% of all the dogs euthanized in 2015 and 2016 [12]. One particularly revealing study, for instance, found that dogs described as “pit bulls” averaged over three times longer stays in shelters than their phenotypically similar lookalike matches who were labeled as other breeds [13].

Scholars and journalists typically trace prejudice against PBTDs back to the rise of negative portraits in media and pop culture during the late twentieth century [1,14,15,16,17,18,19]. Those depictions were described as a veritable “pit bull panic” [20], in which the media regularly ran high-profile sensationalized stories of PBTDs’ involvement in fatal attacks and illegal dogfighting rings [21]. Scholars have also documented the ways in which negative associations between “pit bulls” and Black Americans were propagated by news coverage that heightened racialized fears about crime and threats to public safety [10,17,22,23,24,25,26,27]. These portrayals often characterized ownership of PBTDs as a sign of social deviancy with thinly veiled racial and socio-economic overtones [14,17]. The upshot, as Guenther [27], (pp. 155) surmised, was that perceptions of “pit bulls” changed from “trusted guardians of white children” before the panic era to “*fearsome menaces, an extension of the racist specter of the dangerous, criminal Black man*” by the end of it.

Those negative portraits of “pit bulls” (and their owners) contributed to the emergence and diffusion of over one thousand local laws and ordinances restricting or banning PBTDs from certain localities across the U.S. since 1984. That year, the Village of Tijeras, New Mexico passed the first city ordinance in the United States banning all “pit bulls” and allowing any such dog to be destroyed (Tijeras Ordinance No. 32, Section VII, Paragraph 1. 1984) [28]. After a nine-year old girl in the area was seriously injured by her grandparents’ American Pit Bull Terriers, local news stories repeatedly labeled “pit bulls” as vicious, with one story including a quotation that “pits are designed for killing” [28]. The Village Council then parroted those reports both when enacting the ordinance and in justifying its breed-specific legislation (BSL) to the courts, stating: “*the American Pit Bull Terrier breed possesses inherent characteristics of aggression, strength, viciousness and unpredictability not found in any other breeds of dog*” (emphasis added, *Garcia v. Village of Tijeras*, 767 P.2d 355, 359 (N.M. Ct. App. 1988)) [29].

Both the Tijeras ordinance and its justifications for the law quickly spread throughout the United States. The cycle of a rare but high-profile attack, followed by sensational media coverage that trafficked heavily in stereotypes of “pit bulls” as naturally aggressive and inherently vicious, were common in the 1980s and 1990s. Government officials then cited those socially constructed narratives as fact in their rationales for enacting BSL against “pit bulls” [17,30,31]. Cities and counties—big and small—responded to this supposed public health crisis with alacrity; prohibitions were often treated as emergency orders taking effect immediately. Many of those ordinances specified that failure to comply with all restrictions would result in the “humane killing” of dogs identified as “pit bulls.”

Those prohibitions frequently cast wide nets that were challenged in the courts on the grounds that “pit bull” bans should be “void for vagueness” because no such breed exists (note: “pit bull” is commonly used as an umbrella term that encompasses PBTD breeds and mixes rather than a specific dog breed). While some municipalities limited their ordinances to purebred American Pit Bull Terriers, Staffordshire Bull Terriers, and American Staffordshire Terriers, most BSL restrictions extended to mixed breed dogs who may contain some genetic makeup of those breeds or appear to have the physical characteristics of PBTDs. Such subjective assessments of who counts as a “pit bull” became another point of contention in the debate over BSL, as a series of studies show that visual identifications of dog breeds are deeply flawed even when carried out by animal shelter employees [32,33,34,35].

### The Rise of Pit Bull Positivity

The negative portraits and narratives that were so often used to justify BSL against PBTDs began to change in the early twenty-first century. The rescue of over 50 PBTDs from Bad Newz Kennels in 2007—the illegal dogfighting ring financed by the Atlanta Falcons’ star quarterback, Mike Vick—marked an important turning point in the portrayal of “pit bulls.” The so-called “Vicktory Dogs’” perseverance and forgiving nature in spite of the horrifying abuse they experienced on Vick’s property, for instance, prompted the upbeat headline on the cover of *Sports Illustrated* (SI), “The Good News out of Bad Newz Kennels.” That cover story used the dogs’ rehabilitation (only one of whom was put down for violent temperament) as “*proof that pit bulls have an image problem*” [36]. In doing so, the piece explained why these dogs are “*among the most people-friendly on the planet*;” and it acknowledged how SI’s infamous 1987 cover of a teeth-bearing “pit bull terrier” snarling beneath the ominous headline “Beware of this Dog” contributed to the panic era’s paranoia by cementing “the dogs’ badass cred.” The subsequent bestselling books written by the article’s author, Jim Gorant [37,38], rehabilitated PBTDs’ images even further by chronicling the lives of Vick’s dogs and providing them with individualized positive narratives. Those portraits, in turn, helped reposition “pit bulls” as victims of criminality rather than accessories to it [27], (p. 161).

Similarly sympathetic stories spread rapidly throughout popular culture after the Vick scandal. While our automated content analysis found that the news media persistently portrayed “pit bulls” unfavorably from 2009 to 2017 [31], there were now several countervailing sources of information disputing these negative depictions. Television shows like *Pit Bulls and Parolees*, *Pit Boss*, and *The Dog Whisperer* all explicitly challenged widespread stereotypes and misperceptions about these dogs. Celebrities, such as Miley Cyrus, Jennifer Aniston, Rachel Ray, David Bautista, Jessica Alba, Tom Brady, Billie Eilish, Ariana Grande, Channing Tatum, Kaley Cuoco, Jon Stewart, and Patrick Stewart, became increasingly visible owners who oftentimes advocated for PBTDs. Sir Patrick Stewart even insisted on having a “pit bull companion,” Number One, when he reprised his legendary Star Trek role as Admiral Picard in 2020 [39].

As important as the entertainment industry has been in changing portraits and narratives of these dogs, it pales in comparison to the influence of social media. Social media has become the single greatest outlet for pit bull positivity over the past fifteen years. Take the “animal lovers’ website,” *The Dodo*, for example. *The Dodo* regularly shares its trademarked “Pittie Nation” videos with an audience of over 12 million Instagram followers and 17 million YouTube subscribers—videos intentionally aimed at showing that “Pit bulls are the most cuddly, loving, goofiest dogs in the world” (quoted from *The Dodo*’s Pittie Nation website). That is just a small drop in the bucket of pit bull positivity on social media, too. In fact, our automated content analyses of Twitter (2009–2017) and Instagram (2013–2017) found that there were more positive posts on these platforms about “pit bulls” than any other dog breed [10,31]. Some of those dogs have become celebrities themselves. The social media accounts for these so-called “pitfluencers” have millions of followers, and their posts are often explicitly aimed at breaking down longstanding stigmas and misconceptions about PBTDs [40].

The surge in such positive portraits coincided with growing advocacy against breed-specific legislation. The popular progressive news aggregator, *HuffPost*, ran dozens of stories in the 2010s that used positive portraits of “pit bulls” to attack BSL. Thousands marched on Washington D.C. in May 2014 to advocate for changes in laws affecting ownership of PBTDs [41]; and several notable organizations have issued strong position statements against breed-specific legislation. This prominent list of BSL opponents includes the American Society for the Prevention of Cruelty to Animals [42], The Royal Society for the Prevention of Cruelty to Animals [43], the American Kennel Club [44], the American Veterinary Medical Association [45], the Australian Veterinary Association [46], the British Veterinary Association [47], the American Dog Breeders Association [48], the Humane Society of the U.S. [49], the United Kennel Club [50], the American Bar Association [51], the Association of Professional Dog Trainers [52], State Farm Insurance [53], the National Animal Care and Control Association [54], the American Veterinary Society of Animal Behavior [45], Best Friends Animal Society [55], the National Black Caucus of State Legislators [56], the Center for Disease Control [57], and the Obama White House [58].

These groups’ position statements typically cite multiple reasons for their opposition. Nearly all of them discuss BSL’s ineffectiveness in enhancing public safety. They also frequently reference the growing body of scientific research, cited above, showing that breed is a poor predictor of a dog’s individual behavior and that most enforcers of BSL are unable to correctly identify dog breeds. But one common theme that unites these organizations’ policy advocacy together with the influx of pit bull positivity on social media are narratives about the unfairness of discriminating against good dogs based upon stereotypes and misperceptions instead of individualized behavior (also known as the “*unfair discrimination frame*”). The American Kennel Club [44], for example, states: “*AKC believes a dog should be judged by its deeds, not its breed and supports reasonable, enforceable, non-discriminatory laws. AKC believes that breed-specific legislation is akin to racial profiling for dogs.*” The National Black Caucus of State Legislator’s [56] anti-BSL resolution similarly asserts, “breed-specific legislation is not based on science or data but is based upon outdated stereotypes and conjecture;” and the Association for Professional Dog Trainers’ [52] position statement proclaims, “*Singling out and publicly demonizing certain breeds as dangerous is unfair, discriminatory, and does an immense disservice to those breeds and the people who care about them*.”

Drawing on insights from the respective social science studies on changes in attitudes and public policy, we argue that this influx of positivity and advocacy should have a powerful impact on opinions and policies towards PBTDs. In keeping with that contention, our analysis of two different series of nationally representative repeated cross-sectional surveys, the first from 2014 to 2018 and the second from 2021 through the end of 2024, shows that public opinion about “pit bulls” is significantly shifting in favor of PBTDs. We also show that voters’ support for ballot measures overturning local “pit bull bans” increased substantially during that same 10-year period. Finally, our analysis of the frames and narratives deployed in recent state and local policy debates shows how growing pit bull positivity helped overturn more than 300 laws against these dogs from 2012 to 2024. We conclude with a brief discussion of how shifts in portraits and opinions of PBTDs will likely continue eroding BSL in the U.S. going forward.

## 2. Theoretical Background and Expectations

A long line of social science research shows that sympathetic portraits in media and popular culture can effectively reduce prejudiced attitudes towards a wide array of outgroups [59,60,61]. One proven avenue through which mediated content can reduce prejudice is by portraying members of marginalized groups in ways that counter societal stereotypes. Vicarious modes of contact are most effective at changing prejudiced attitudes when virtual interactions challenge stereotypes and those positive parasocial encounters are presented as typical of experiences with the whole group [61]—precisely the type of content often found in positive portraits of PBTDs on social media [40,62,63].

Several observational and experimental studies show that this type of sympathetic media coverage can reduce prejudice against immigrants, gays and lesbians, Muslims, working women, racial and ethnic minorities, the mentally ill, interracial couples, and overweight people [64,65,66,67,68,69,70,71,72,73,74,75,76]. There is even some remarkable evidence from field experiments showing that media messages helped reduce deeply ingrained ethnic tensions between Hutus and Tutsis in post-genocidal Rwanda [77,78].

If, as these studies indicate, positive media portraits can effectively reduce prejudice against salient outgroups, then we would certainly expect them to affect public opinion about pit bull-type dogs. After all, biases against human outgroups are oftentimes rooted in group interests, religion, and societal hierarchies. Dominant groups construct prejudice in such circumstances to justify and legitimize the oppression and exploitation of subordinate racial, ethnic, and religious minorities (see [79] for a thorough account). So much so that prejudiced attitudes help structure social orders for generation after generation, as children learn the prevailing norms used to justify existing racial and ethnic hierarchies at young ages [80]. Challenges to those entrenched hierarchies can therefore be quite threatening to dominant groups, making prejudiced attitudes difficult to dislodge.

In stark contrast with those socio-structural accounts, the psychological nature of prejudice against PBTDs is obviously not central to group-based hierarchies, social status, and mass belief systems since humans are not competing with dogs for scarce resources. Some prominent social psychologists have even likened biases against “pit bulls” to a psychological phobia since the cause of their aversion is seen as a characteristic of the person responding to the dog rather than a characteristic of the dog [81], (pp. 156). As such, we suspect that positive contact with PBTDs (both in the real world and vicariously via media) is more effective at reducing prejudice about “pit bulls” than interpersonal and mediated interactions are at changing attitudes about racial, ethnic, and religious groups. Our first empirical expectation, then, is that we should see the recent influx of pit bull positivity translate into greater public support for these dogs over time.

Changes in portraits and opinions should also affect public policies towards pit bull-type dogs. At a minimum, we expect that these positive shifts will lead to substantially stronger support for recent ballot measures—legislative proposals in which citizens voted on whether to repeal their city/county’s ordinance banning PBTDs—than there had been in the past. Much more importantly, though, social science research further suggests that the surge in sympathetic opinions and narratives of “pit bulls” should have a far greater impact on policymaking beyond just the handful of localities where residents have voted on referendums to repeal local prohibitions on PBTDs.

For starters, several political science studies show significant links between public opinion and policymaking. Page and Shapiro’s [82] classic study of national policy in the U.S. from 1935 to 1979 found considerable congruence between changes in mass preferences and changes in public policy (see also [83,84]). Caughey and Warshaw’s [85] analyses from 1936 to 2020 similarly show that state policymaking generally responds to public opinion over time (see [86,87] for additional evidence of state-level policy responsiveness). There is even experimental data showing that state legislators who were randomly assigned to receive info about their district’s preferences were much more likely to vote in line with constituent opinion than those who did not [88].

To be sure, the correspondence between public opinion and policy is far from perfect, especially when the majority’s policy positions conflict with the economic interests of big businesses and wealthy Americans [89,90,91,92]. Policy responsiveness also varies considerably across political institutions and issues [85,86,92,93]. But there is solid evidence that public opinion exerts at least some influence over policy outcomes (see [93,94] for reviews).

Schneider and Ingram’s [95,96] influential work on the social construction of target populations is even more important for our purposes of understanding how changes in portraits and opinions of PBTDs have affected policymaking. The social construction of target populations “refers to the cultural characterizations or popular images of the persons or groups whose behavior and well-being are affected by public policy” [95], (pp. 334). This theory contends that the socially constructed stereotypes of specific groups in positive (“advantaged”) or negative (“deviant”) terms has a powerful influence on public officials and policy, as these images and narratives provide important rationales to legitimate politicians’ goals and decisions.

Several empirical, qualitative and mixed-method studies support Schneider and Ingram’s argument by showing that policymakers are more likely to adopt innovations that extend benefits to popular target populations and impose burdens on marginalized groups (see [97,98] for reviews). This includes animal rights policy, where the public’s more positive socially constructed perceptions (measured in a national survey) of birds, mammals, and fish (advantaged) than reptiles, amphibians, invertebrates (deviants) closely correspond with the policy benefits these species received from the U.S. Endangered Species Act [99] (see also [100] for a detailed analysis of the social construction of narratives in animal welfare discourse and debates).

A series of qualitative analyses have used social construction frameworks to explain the causes and consequences of portraying PBTDs (and their owners, e.g., [101,102]) as vicious deviants who threaten public safety [18,103,104,105,106]. Those accounts explain how the negative portraits and narratives that were propagated by media sensationalism that often trafficked in anti-Black stereotypes during the 1980s and 1990s shifted perceptions of “pit bulls” from favorite companion animals at the beginning of the twentieth century to locked-jawed societal menaces by the end of it. This research is essential to understanding the emergence and diffusion of over 1000 breed-specific regulations on PBTDs across the United States. But there is a dearth of academic research on how the recent rise in pit bull positivity has affected public policy.

Once again, Ingram and Schneider [107] provide key insights here that inform our theoretical expectations. “*For substantial change in the social construction of a target population to take place*,” they explain, “*the frame, discourse, and narrative also must change in a persuasive way*.” This same process seems to be happening now with the rise in positive portraits of PBTDs in social media and popular culture. Or, as one pitfluencer’s parent put it, “*When social media wasn’t what it is today, all we had were sensationalized reports about pit bulls mauling children and dogfighting. This kind of puts the power back in our hands to change that narrative*” (quoted in [40]).

If the social construction of “pit bulls” is indeed shifting from dangerous deviants who need to be regulated (e.g., the public safety frame) to more sympathetic narratives of PBTDs as victims of unwarranted discriminatory policies (e.g., the unfair discrimination frame), then those changes should be reflected in both public opinion and policy. We, therefore, expect to see a significant erosion of laws targeting “pit bulls” in the past decade; and we should also see politicians’ justifications for overturning BSL echo the changing narrative of PBTDs as generally good dogs who are unfairly stereotyped as inherently aggressive and often unjustly subjected to discriminatory policies that judge them by their breeds and not their deeds.

## 3. Methods and Materials

This study uses a variety of different methods and materials to interrogate our theoretical expectations. To test our first hypothesis that support for PBTDs is on the rise in the United States, we draw on a series of nationally representative surveys conducted by the highly ranked survey firm, YouGov (see [108] for pollster ratings), from 2014 up through the end of 2024. Ideally, we would be able to test this “growing support hypothesis” by comparing recent attitudes about “pit bulls” to public opinion before Mike Vick’s 2007 dogfighting scandal helped usher in a new era of positive portraits; but unfortunately, the earliest national survey on “pit bulls” in the United States that we are aware of was conducted by YouGov for the *HuffPost* in July 2014 [109]—several years after portraits and images of PBTDs began changing.

Even though our analyses are probably a conservative estimate of how much attitudes have changed over the past three decades, we can still glean important insights into public opinion dynamics by re-asking some of the exact same questions that first appeared on that 2014 YouGov/HuffPost survey. To ensure an apples-to-apples comparison of repeated cross-sectional data from the same polling firm, we included four questions from the 2014 poll on another survey conducted by YouGov, the 2018 Cooperative Congressional Election Survey (CCES, [110]). Our team module for the CCES asked a nationally representative sample of 1000 adults the following questions in October 2018: (1) if it should be legal or illegal to own a “pit bull”; (2) if “pit bulls” are naturally more aggressive than other breeds; (3) if it is safe or too dangerous for “pit bulls” to live in residential neighborhoods; and (4) if the respondent would personally consider adopting a “pit bull.”

Unfortunately, we do not have additional data on those four questions after our 2018 CCES. But YouGov has fortuitously measured the popularity of over 50 different dog breeds in its quarterly tracker surveys from April 2021 up through the end of 2024. These tracker surveys first asked nationally representative samples (sample sizes range from 1190 to 5709 U.S. adults per quarter) if they have heard of a particular dog breed. The tracker surveys then asked respondents whether they like, dislike, or feel neutral toward the breed. In YouGov’s Q4-2024 tracker survey of 4026 adults, for instance, 56.5% said they liked the American Pit Bull Terrier, compared to 15% who disliked them, 22% who felt neutral, and 6.8% who had not heard of the breed [111]. We use these trackers not only to test our expectation that support for PBTDs is growing, but to show how these changes in attitudes about the American Pit Bull Terrier compare to shifts in views of other well-known dog breeds.

We then employ multiple methods to help triangulate our expectation that shifts in opinions, portraits, and narratives of “pit bulls” have undermined BSL across the U.S during the past decade. The most straightforward analysis here compares support for ballot measures in which residents voted on repealing their local government’s “pit bull ban” in 2012 and 2014 to election returns from 2018 to 2024 to test our expectation that voters are now more supportive of repealing BSL targeted at “pit bulls” than they were in the past.

Our analysis of local ballot measures pays particularly close attention to the narratives and discourse surrounding pit bull politics in Aurora, Colorado. Aurora offers unique insights into changes in both mass and elite support for banning these dogs from the state’s third-largest city. Indeed, the Aurora city council’s 2005 ban on PBTDs (City Code Section 14–75. Ordinance No. 2005-84) [112] was upheld by the courts in 2009, reaffirmed by voters in the 2014 general election, repealed by the city council in 2021, reinstated by the courts in 2024, repealed by voters in the November 2024 general election, and then officially overturned by the city council in December 2024. We, therefore, analyze city council hearings, news coverage, court rulings, and campaign content from Aurora as a case study to interrogate our expectation that pit bull positivity and unfair discrimination frames are increasingly present in the socially constructed narratives used by politicians and the public to justify repealing breed-specific legislation. We then map out the geography of over 300 BSL repeals enacted from 2012 to 2024 to support our expectation that the changes in narratives, which helped repeal Aurora’s ban, are occurring on a broader scale throughout much of the United States.

This article’s remaining analyses shift our focus from local ordinances to statewide policies. At the time of this writing, nearly half of the states have passed preemption laws that prevent local governments from enacting/enforcing BSL banning specific dog breeds [113,114]. We analyze legislative debates, news coverage, and campaign content from the latest state to do so, Florida, to help identify the frames and narratives that are now used to justify these laws. Like Aurora, the distinctive state, local, and electoral politics surrounding Miami-Dade County’s 1989 ban on PTSDs (County Code 5-17. Ordinance No. 89-22) [115] makes Florida a particularly informative case study.

## 4. Results and Analysis

### 4.1. Shifts in Public Opinion, 2014–2024

**Figure 1:**
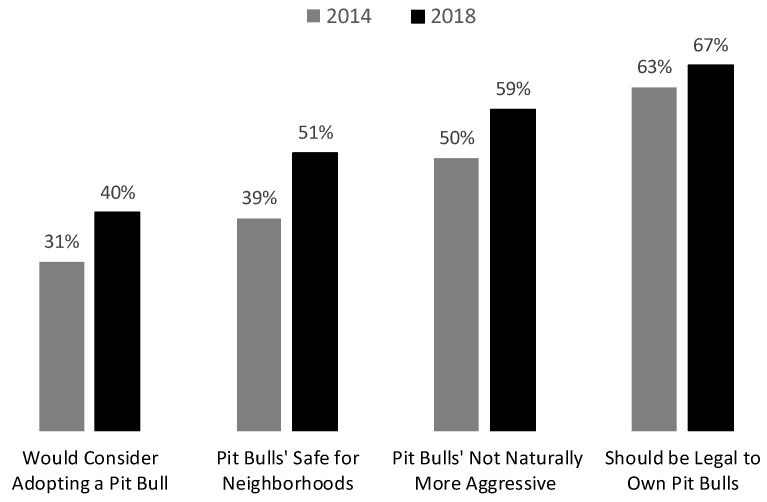
Changes in Public Opinion About Pit Bulls from 2014 to 2018. Sources: HuffPost/YouGov Survey July 2014. CCES/YouGov Survey, October 2018.

Figure 1 tests our first expectation about growing public support for “pit bulls” by showing how responses to YouGov’s four questions about these dogs changed from 2014 to 2018. You can see that answers to all four questions moved in support of “pit bulls” during that period. Those shifts ranged from a modest four-percentage-point increase in support of legalization to a substantial 12-point jump in the share who said it us “safe for pit bulls to live in residential neighborhoods.” The differences in percentages from 2014 to 2018 for the first three questions in Figure 1 are all highly significant (*p* < 0.0001), while the four-point difference in support for legalization shown in the final column of the figure is marginally significant (*p* = 0.06).

Averaging across the four different items, there was an 8.5 percentage-point shift in support of pit bulls from 2014 to 2018. This 8.5-point difference in mean support from 2014 to 2018 is also highly significant, with the 95% confidence interval for that estimate ranging from 7.4 to 9.6-points (T-score = 14.9, *p* < 0.00001). That significant shift is even more remarkable when considering how depictions of PBTDs in popular culture had already changed by July 2014. Our baseline levels of public support for these dogs would have almost certainly been lower had there been survey data available from the early 2000s before the new era of pit bull positivity began.

**Figure 2:**
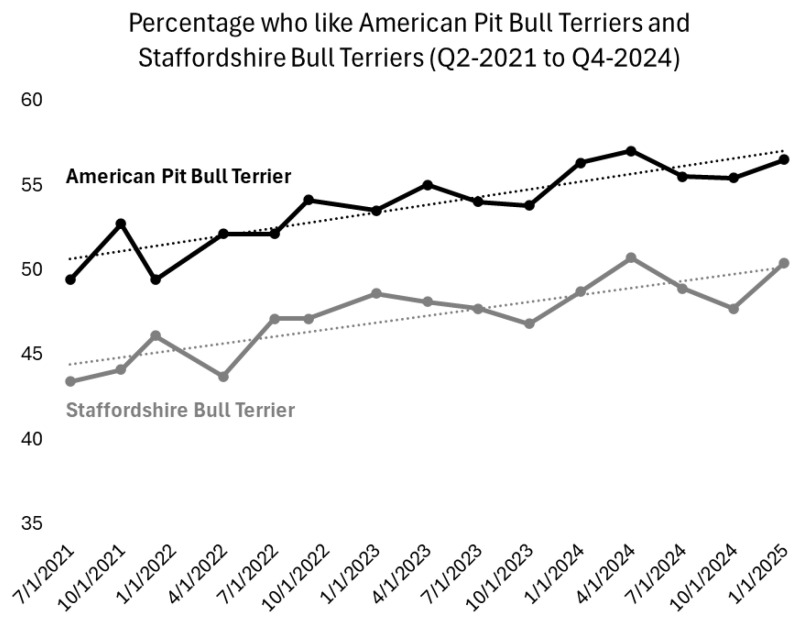
Likability Ratings of American Pit Bull Terriers and Staffordshire Bull Terriers from Q2-2021 to Q4-2024. Note: Linear trend lines produced from OLS regression coefficients in Table 1. Source: YouGov Quarterly Tracking Surveys.

The results in Figure 2 further bolster that conclusion by showing that public support for PBTDs has continued to steadily increase since 2018. The black dots in Figure 2 show the share of respondents per quarter who said they liked the American Pit Bull Terrier (APBT) in YouGov’s [116] quarterly tracking surveys. Meanwhile, the gray points show the share of respondents who said they liked the other dog breed that is most frequently labeled as a “pit bull,” the Staffordshire Bull Terrier (“Staffy;” [117]). The APBT’s higher likeability scores in the figure stem from the fact that far more Americans have heard of this breed than the Staffy. On average, 92.9% recognized the American Pit Bull Terrier in the 2021–2024 surveys, compared to just 79.4% who knew of the Staffy.

But you can see that the upsurge in support for both PBTDs is quite similar, nonetheless. The black line in Figure 2 shows that the share of adults who liked the American Pit Bull Terrier steadily increased from 49.4% in Q2-2022 to 56.5% by the end of 2024. That 7.1-point difference in percentages is highly significant, too, with the 95% confidence interval for this estimate ranging from 3.8 to 10.8-points (*p* < 0.0001). The gray line shows that the Staffy’s popularity also increased by a highly significant 7-points from 43.4% who liked them in 2021 to 50.4% at the end of 2024 (95% confidence interval for difference in proportions = [3.7 to 10.3-points], *p* < 0.0001; note: since information is not available on the sample size of YouGov’s Q2-2021 quarterly tracker, our analyses conservatively use the smallest sample size in the entire quarterly tracker series for Q2-2021 (1190) in our confidence interval estimates).

Table 1 further shows that the regression coefficients, which produced the two dashed linear trend lines displayed in Figure 2, are both highly significant as well (*p* < 0.0001). Those linear regression coefficients indicate that the share of respondents who liked the American Pit Bull Terrier increased by nearly half of a percentage point every quarter from July 2021 through December 2024, and that the Staffy’s popularity grew about four-tenths of a point per quarter. The R^2^ figures at the bottom of Table 1, meanwhile, show that time (i.e., the quarter when the survey was conducted) alone explains around 70% of the variation in quarterly support for the Staffy and 76% of the variation in the APBT’s likability scores from Q2-2021 to Q4-2024.

**Figure 3:**
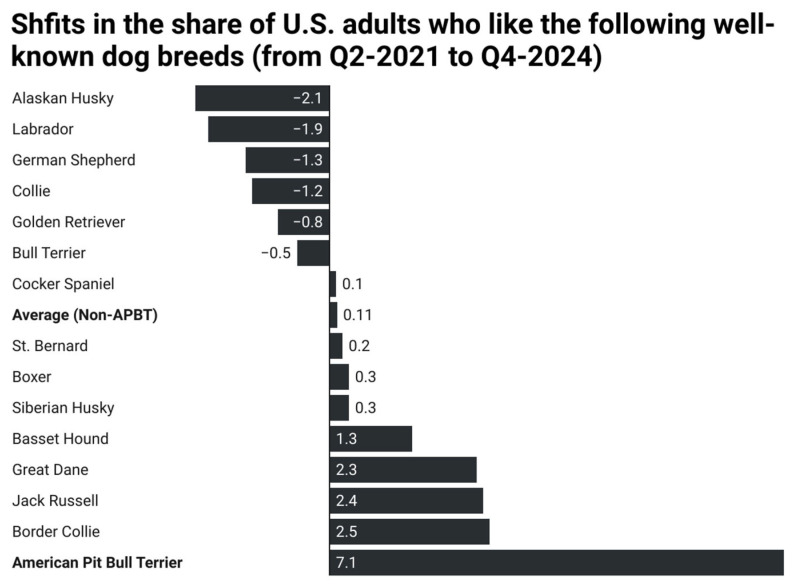
Changes in the Popularity of America’s Best-Known Dog Breeds (from Q1-2021 to Q4-2024). Note: Change scores calculated by subtracting the percentage who liked the dog breed in Q4-2024 from the percentage who liked them in Q2-2021. These were the 15 best-known dog breeds according to data from YouGov’s Q4-2024 tracker survey. Source: YouGov Quarterly Tracker Surveys.

The growing support for American Pit Bull Terriers shown in Figure 2 and Table 1 is even more striking given the nonsignificant changes in opinions about other equally well-known dog breeds from 2021 to 2025. You can see that stark contrast in Figure 3, which shows shifts in overtime support (from Q2-2021 to Q4-2024) among the fifteen best-known breeds in YouGov’s Q4-2024 tracker (all fifteen were recognized by at least 91% of respondents). The display shows virtually no change whatsoever (only one-tenth of a percentage point on average) from 2021 to the end of 2024 in the mean share of U.S. adults who said they liked these other 14 dog breeds. That obviously pales in comparison to the highly significant 7.1-point shift in support for the American Pit Bull Terrier.

Finally, it is important to conclude this section on growing popularity by noting that the same factors shifting public opinion in support for “pit bulls” appear to have also produced an enormous generational divide in attitudes about these dogs. Young adults under the age of 25 in the CCES were 40–60 percentage points more supportive of the four positions in Figure 1 than senior citizens. In fact, age was consistently the strongest predictor of Americans’ attitudes about “pit bulls” in a series of multivariate analyses presented elsewhere—analyses that modeled opinions of these dogs as a function of age, race, racial attitudes, party identification, gender, and education [10]. It is not simply the case that young people just like all dogs more than their older counterparts, either. Our related research shows that age is an equally powerful predictor of which Americans have more favorable views of well-known dog breeds than “pit bulls” (Ibid, Table 1).

These sizable age effects in U.S. surveys show up in polling data from other countries as well. A 2023 YouGov-UK survey, for example, showed British seniors were 46-points more likely to support the UK’s ban on the American Pit Bull Terrier than young adults under the age of 25 (76% to 30%, respectively). Similarly, an October 2016 poll by Forum Research for the Montreal Gazette found that senior citizens were 35-percentage points more likely to support the French-Canadian city’s “ban on pit bull-type dogs” than younger adults under the age of 35 (62% to 27%). And prior research found that age was a strong predictor of support for banning “pit bulls” in the Bahamas [118].

The large generational divide in public opinion about “pit bulls” falls perfectly in line with social science research showing that when societal depictions of a group shift, those changes are usually first adopted by adolescents and young adults. Social and political attitudes are simply more malleable during our “impressionable years” of adolescence and early adulthood than they are later in life [119,120,121], making young people most likely to embrace society’s shifting norms about specific groups [122,123]. These findings, therefore, suggest that public support for PBTDs should continue to grow as older Americans, who disproportionately dislike “pit bulls,” are increasingly replaced in society by younger generations whose more favorable opinions of these dogs were formed in the new era of pit bull positivity.

### 4.2. Repealing Local BSL

**Figure 4:**
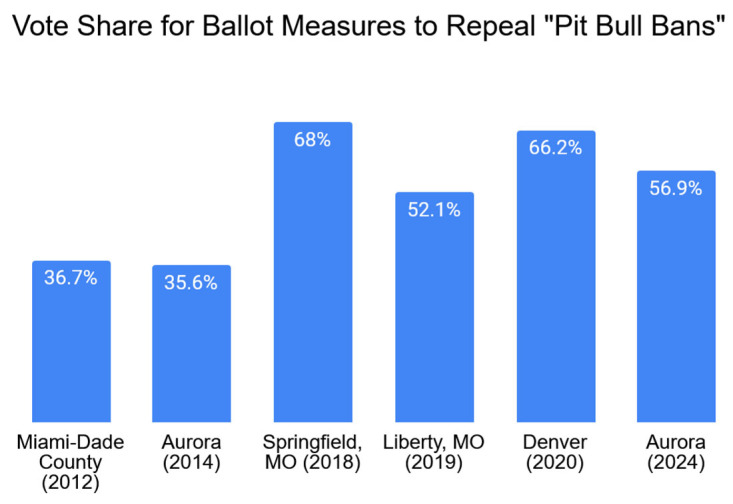
Election Results for Ballot Measures to Overturn Local Bans on “Pit Bulls”, 2012–2024. Source: Official Election Returns from city/county.

If, as we suggested, changes in portraits and opinions of “pit bulls” are affecting policies toward PBTDs, then there should at the very least be greater support for ballot initiatives—legislative proposals in which residents voted on repealing BSL—than there had been in the past. Figure 4 tests that expectation by displaying the results from the six ballot measures we are aware of in which residents voted on repealing their city/county’s BSL bans on “pit bulls.” The two left-hand columns of the figure show that the unsuccessful efforts to repeal Miami-Dade’s and Aurora’s “pit bull bans” in 2012 and 2014 were only supported by approximately 36% of voters in those respective areas. The final four columns of the display, however, show that ballot measures to repeal citywide “pit bull bans” from 2018 to 2024 were, on average, supported by over 60% of voters.

While these results are certainly consistent with our expectations of growing voter support for repealing local BSL bans at the ballot box, there is only so much you can learn from comparing election results across different localities. Even comparing neighboring cities, such as Denver and Aurora, is complicated by underlying differences in demographics and sociopolitical attitudes. As such, the within city comparison between Aurora’s 2014 and 2024 ballot measures in Figure 4 is particularly important. You can see that Aurora voters were over 20 percentage points more supportive of repealing the city’s ban on PBTDs in 2024 than they were in 2014. That sizable shift is generally in line with the combined national trends in public opinion from Figure 1 and Figure 2 during that same ten-year period; and it is by far the strongest evidence showing greater overtime support for ballot measures that repeal local bans on PBTDs.

In addition to showing voters’ stronger support for repealing BSL at the ballot box, the singular circumstances surrounding pit bull politics in Aurora provide key insights into the evolving frames deployed in electoral, judicial, and legislative debates about the policy. The city council passed its ban on PBTDs in 2005, soon after a “pit bull mauled” eleven-year-old Xavier Benavidez in an unprovoked attack (quoted from [124]). It is not surprising, then, that the city relied heavily on negative portraits and narratives of “pit bulls” as threats to public safety in justifying the ban.

So, too, did the court when it upheld Aurora’s BSL ban in 2009. The American Canine Foundation sued the city, arguing that the ban violated the Due Process and Equal Protection Clauses of the 14th Amendment. But the court denied any constitutional violation, finding instead that Aurora had a rational basis for exercising the city’s police power to protect public safety. In doing so, the ruling drew heavily on negative narratives and stereotypes that “*there was a fighting propensity among pit bulls and that they were more aggressive than other dogs*.” The decision goes on to state as a matter of (unsubstantiated) fact, “*Most dogs will growl or bark as a warning, pit bulls do not. In fact, pit bulls can be wagging their tails and instantly go into an attack* (*American Canine Foundation v. City of Aurora, Colorado,* Civil Action No. 06-cv-01510-WYD-BNB. 8 May 2009)” [125].

The next development in the Aurora saga came five years later when the issue appeared on the ballot. The 2014 referendum on “pit bulls” captures an interesting juncture in the emerging shifts in attitudes and narratives about PBTDs. As one news story noted, there were around 700 cities with bans on PBTDs at the time, but “*Pit bulls are getting a warmer reception in recent years.*” [126]. Those increasingly positive narratives of PBTDs as generally good dogs who are unfairly subjected to discriminatory laws were frequently deployed in the campaign by supporters of Measure 2D. The Facebook page for ColoRADogs, a non-profit group leading the charge to repeal the Aurora ban, even likened their advocacy with efforts “*seeking equal rights for gay, lesbian, and transgender people*” (quoted in [124]).

But the proposition’s opponents were quick to counter the unfair discrimination frame with negative narratives of PBTDs as dangerous threats to public safety. Supporters of the ban ran multiple advertisements in two local papers, the *Denver Post* and *The Sentinal* stating, “*Fact: pit bulls kill more humans and animals than all breeds combined.*” While the *Denver Post* temporarily pulled the advertisement for not substantiating its claim, *The Sentinal* strongly supported the city’s ban and had no such misgivings. In an October 2014 editorial, the paper’s staff urged readers to vote no on Measure 2D, leaning heavily into public safety frames by attacking arguments that PBTDs are no more dangerous than other dogs as “*pseudo-science and irrelevant studies that these misguided pit-bull aficionados continually trot out*” [127]. Nearly two-thirds of voters sided with that position in rejecting the referendum.

Aurora citizens and animal rights advocates continued their fight against the ban after the resounding electoral defeat. In reaction to those efforts, the city council began debating whether they should put the issue back on the ballot or even repeal the ban entirely themselves. In the council’s first debate over the issue on 17 August 2020, Councilwoman Bergan said that she believed “*public opinion has changed since 2014. I think it’s probably favorable to the repeal (emphasis added), but I do think the question should go back to the voters*” (Aurora City Council meeting, 17 August 2020). When the issue returned to the agenda in December 2020, there was a clear divide between those that framed the issue in terms of discrimination versus those that adopted the public safety frame. Councilwoman Coombs, for instance, spoke of the need for the council, not voters, to address the fact that “*breed discrimination has been written into our code*” (Aurora City Council meeting, 21 December 2020). The other younger members of the council agreed, as Councilman Marcano argued “*it was created by the council. It should be undone by the council*” (Ibid).

In keeping with the large generational divide over PBTDs, an older member of the council who had voted for the ban back in 2005 recounted her reasoning for doing so: “*We saw with our own eyes*,” Councilwoman Berzins recalled, “*and heard from people about the dangers and the mauled children—the children that have lost their faces and arms, had their stomachs ripped open*” (Ibid). When the issue was raised again the following month, she went on to warn her colleagues of the repercussions of repealing the ban, stating: “*For those of you that vote for this, just remember that you did. And when a horrible accident happens, just remember that you voted to have them [PBTDs] back in the city… it’s not a dog issue, it’s a public safety issue*” (Aurora City Council meeting, 21 January 2021). Most of the council rejected this public safety frame, though, repealing the ban by a vote of 7 to 3.

After a district judge ruled that the council’s unilateral repeal of the ban on PBTDs violated the will of the people, the issue was once again put on the ballot in November 2024. Question 3A met a much different fate from the 2014 proposition, passing with 56.1% of the popular vote. The resulting legislation echoed shifting narratives that dogs should be judged by their deeds, not their breeds, reserving vicious dog restrictions only for canines who have actually engaged in violence. Indeed, the campaign in support of 3A regularly deployed these frames, arguing “breed-specific bans create a stigma around pit bulls,” and they should instead be replaced by “behavior-based legislation that penalizes individual aggressive dogs rather than entire breeds” (Quoted in [128]).The group Stop Aurora Pit Bull Ban (now called Equality for All Breeds), informed the public that “By voting YES [on Question 3A], you’re saying that all dogs deserve to be judged individually, based on their behavior and upbringing, not simply by how they look” (See statement here: https://stopaurorapitbullban.com/, accessed on 30 June 2025). Similarly, End Aurora BSL, informed voters that the “various breeds considered ‘pit bulls’ are held to a higher standard than most dogs, receiving far more scrutiny for often normal dog behaviors” (See statement here: https://endaurorabsl.org/, accessed on 30 June 2025).

One month after the election, the Aurora city council codified voters’ decision into law. The ordinance amending the city code pertaining to breed-specific restrictions passed unanimously. In talking about the long and winding road to reach that point, members of the council praised animal advocacy groups and dog lovers for their unwavering campaign to educate the public about PBTDs. Councilwoman Jurinsky noted that proponents “really worked hard on this. Knocked on doors, created flyers, went from pet store to pet store” (Aurora City Council meeting, 2 December 2024). Councilwoman Coombs opined that the repeal’s success was an example of the power of the people. She credited “pit bull” advocates for continuing to speak up “until it became an agenda item” (Ibid). Simply put, the city council ultimately responded to shifting portraits and opinions of pit bull-type dogs.

**Figure 5:**
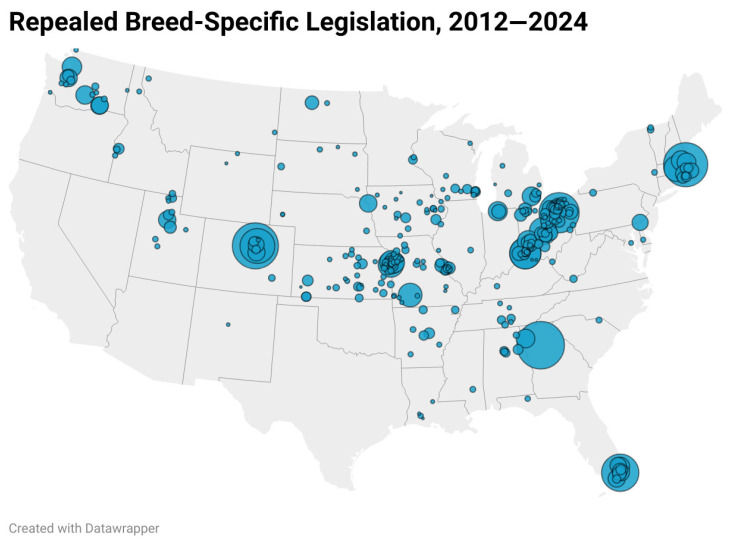
Map of Local BSL Repeals by Population Size, 2012–2014. Note: States with laws preempting local BSL (without exceptions) before 2012 are Texas, Pennsylvania, New York, New Jersey, Illinois, Virginia, Minnesota, Maine, and Oklahoma. Source: National Canine Research Council (2025).

Figure 5 suggests that these changes in opinions and narratives, which helped repeal Aurora’s ban on PBTDs, have also undermined breed-specific legislation throughout much of the United States. This map of BSL repeals from 2012 to 2024 reveals a couple of noteworthy patterns. Most importantly, you can see the large number of BSL repeals represented by the population-scaled circles on the map. Those dots show that over 300 breed-specific laws and ordinances were repealed from 2012 to 2024 in cities/counties that have a combined population of more than nine million people. The 300+ BSL repeals shown in Figure 5 account for over a quarter of the local breed-specific laws that were in place beforehand. By contrast, we identified only five cities, with a combined population of fewer than 14,000 residents, which enacted more stringent breed-specific regulations against PBTDs since 2019.

The conspicuous geographical clustering on display in Figure 5 is also important. Some of that proximity is simply a byproduct of new statewide laws preempting local governments from enacting/enforcing BSL in Massachusetts (Chapter 140, Section 157), Connecticut (Chapter 98 Section 7-148(D)(i)), Rhode Island (§4-13-43 and §4-13.1-16), Utah (18-2-1), Washington (Chapter 16.08,110 RCW), and Florida (Section 767.14-2023). Meanwhile, the absence of BSL repeals in many western states stems from the fact that California, Oregon, New Mexico, Nevada, and Arizona had only a few breed-specific regulations to begin with. The clustering of repeals in nearby cities across the midwestern states without preemption laws, however, is consistent with policy learning models in which states tend to adopt the same policies as their neighbors [129]. The patterns in the map are also consistent with Fix and Mitchell’s [113] analysis of the significant role of geographical proximity in the diffusion of BSL preemption laws across the states. It therefore appears that changing narratives and opinions of “pit bulls” have helped produce a domino effect on policy in which their role in repealing one municipality’s BSL then spreads to nearby cities.

### 4.3. Policy Narratives and State Laws Prohibiting Breed-Specific Discrimination

Like Aurora, the distinct circumstances surrounding BSL in Florida offer important insights into how the frames and narratives deployed in pit bull policy debates have changed since Miami-Dade banned PBTDs from the state’s most populous county in 1989. County commissioners passed the ban soon after a seven-year-old girl, Melissa Moreira, was seriously injured by a stray “pit bull.” As was so often the case in the pit bull panic era, county officials used this attack as evidence that PBTDs are inherently dangerous threats to public safety who should be banned from the area [130]. The following year, Florida became one of the first states to pass a preemption law that prohibits local governments from enacting BSL (Section 767.14); but this 1990 law grandfathered in breed-specific restrictions already in place such as the Miami-Dade ordinance.

The emergence of new narratives and advocacy helped put the issue back on the state’s legislative agenda in 2012. Those efforts were further aided after the Miami Marlins’ newly acquired all-star pitcher and “pit bull parent,” Mark Buehrle, had to move his family to Broward County because of the ban. But a group of Miami-Dade commissioners short-circuited what the *Miami Herald* described at the time as an apparent “winning effort in the state legislature to abolish the ban” with its April 2012 decision to put the issue on the ballot in the statewide primary election taking place in August [131]. Anti-BSL organizations were outraged over the alacrity with which the county commission put the issue up for a vote. Or, as the founder of the Miami Coalition Against Breed-Specific Legislation (MCABSL) put it, “We only had a few months to reeducate an entire county that has been brainwashed for 23 years” (quoted in [131]).

Advocates, such as MCABSL, Best Friends Animal Society, Shorty Rossi from the show *Pit Boss*, and the Buehrles, launched a valiant campaign to do so. The Buehrles’ Staffy, Slater, was even described by the *HuffPost* as “the face” of the campaign since positive portraits of his loving nature and gentleness with children were frequently used to highlight the unfair discriminatory nature of the Miami-Dade ban (quoted in [132]). Those images were contrasted with Moreira’s still-scarred face and her strong support for the BSL that was enacted shortly after she was attacked back in 1989. She recounted her fears to local media during the campaign that voters would repeal the ban and “another kid was going to go through what I went through” (quoted in [133]). Despite her fears that the resolution would succeed, Figure 4 shows that over 60% of the county voted in favor of keeping the ban in place.

The campaign to end BSL in Florida nevertheless continued, as Mark Buehrle and his fellow advocates took the defeat as evidence that they needed to do more work “to educate about the ‘pit bull-type dog’ breed” (quoted in [131]). Those same educational efforts were, of course, taking place on a much larger scale nationwide thanks to the rise of pit bull positivity in social media and popular culture since 2012. So, it is not surprising that lawmakers were more receptive to ending the ban when the issue returned to the Florida legislature’s agenda ten years later. Senator Garcia, the author of the 2022 bill to end all local breed-specific laws in the state (SB 614) [134], drew heavily on the unfair discrimination frame in telling the chamber’s agricultural committee that “*certain incidents have magnified this (vicious behavior) to a particular breed such as pit bulls… There isn’t any scientific evidence that there is a particular breed that is dangerous… there are incidents, but the good ones pay for the bad*” (Florida Senate Agricultural Committee meeting 10 February 2022).

Multiple members of the Florida legislature also drew on their own positive experiences with “pit bulls” to portray them as loving dogs who are unfairly tainted by stereotypes. When presenting the bill, for example, Representative Buchanan joked about how he wanted to bring his PBTD, “Fluffy,” in for show-and-tell (Florida House Regulatory Reform Subcommittee meeting, 8 February 2022). Representative Chaney similarly stated that she has “*a pit bull mix dog and she’s the sweetest dog I’ve ever known*” (House Local Admin and Veteran Affairs Subcommittee meeting, 11 January 2022). The chair of the committee, Representative Toledo, thanked Buchanan on behalf of his dog “*for bringing this dog discrimination bill forward*” (Ibid). These debates and narratives set the stage for a similar bill (HB 941/SB 942) [135,136] to fly through the various steps of the legislative process in 2023 with little to no debate or resistance. So much so, in fact, that the once-contentious issue fell just one vote shy of unanimously passing through both chambers of the Florida legislature (155-Yeas to 1-Nay). The legislation overturning Miami-Dade County’s 34-year-old ban on PBTD’s was officially signed into law by Governor Ron Desantis in June 2023.

While the new Florida law prohibited all local governments and public housing authorities in the state from banning dogs based on their breed, size, or weight, it still allowed private entities, such as landlords and insurance companies, to discriminate against pit bull-type dogs. The next frontier in breed-specific policy debates is, in fact, increasingly waged in state legislatures over bills that forbid property insurance companies from denying coverage to owners of certain dog breeds (most commonly, PBTDs). Nevada and New York became the first two states to sign these policies into law in 2021; and Illinois and Pennsylvania followed suit in 2023. At least nine other states are considering similar legislation at the time of this writing.

The justifications for enacting these laws are driven in large part by pit bull positivity and unfair discrimination frames. Nevada state senator, Melanie Scheible, said in support of the legislation she sponsored: “*I would like to see more people being open to adopting a pit bull or a Rottweiler from the shelter; because these dogs are no more likely to be dangerous than any other dog*” (quoted in [137]). Senator Bob Duff, who co-sponsored a similar bill in Connecticut, described his three PBTDs as “the best dogs. They’re just fun” (quoted in [138]); and State Farm [53] defended its support of anti-discrimination insurance policies by stating: “*Just like humans, dogs are individuals. Every dog has a unique personality. While a dog’s breed may dictate what the dog looks like, how a dog reacts to people or situations isn’t guaranteed by breed or type*.”

It appears, then, that the rise in positive portraits, narratives, and opinions of “pit bulls” is not just undermining BSL throughout much of the United States. Pit bull positivity is also spilling over into state legislation aimed at extending greater anti-discrimination protections to owners of PBTDs by preventing private entities like insurance companies from enacting discriminatory policies against these dogs.

## 5. Discussion

The findings in this article, which showed that changing portraits and opinions of “pit bulls” helped erode breed-specific legislation in the United States, contribute to the academic literature on public opinion, prejudice reduction, public policy, and animal studies in a variety of ways. First, our results provide a novel example of social media’s ability to affect public opinion about animals in meaningful ways that can, in turn, change public policy. If anything, the magnitude of the attitudinal changes presented in Figure 1, Figure 2 and Figure 3 suggest that media messages are even more effective at reducing prejudice towards stigmatized animals than marginalized groups of people, presumably because biases against human outgroups are oftentimes deeply rooted in group interests, religion, and societal hierarchies. Social media may, therefore, offer an especially important avenue for animal rights advocacy. Our results also contribute to the growing body of research on how the social construction of animals in general, and of “pit bulls” in particular, affects policy outcomes towards them. These analyses suggest that the socially constructed narratives surrounding various animals are not necessarily fixed. Instead, the emergence of BSL stemmed in large part from shifting narratives of “pit bulls” from favorite companion animals at the beginning of the twentieth century to deviant menaces by the end of it. Those negative narratives were then countered by the rise of pit bull positivity and socially constructed narratives of PBTDs as good dogs who are unfairly subjected to discrimination—frames that undermined support for BSL policies over the past decade.

This, of course, does not mean that changing images and opinions of “pit bulls” are *the only* reason why hundreds of local breed-specific restrictions were repealed in the past decade. There are rarely monocausal explanations for policy changes, and other factors have inevitably interacted with pit bull positivity to undermine BSL in the USA. We noted earlier, for instance, that a growing body of scientific research shows that BSL is ineffective in enhancing public safety, that breed is a poor predictor of a dog’s individual behavior, and that those tasked with enforcing “pit bull” bans are oftentimes unable to correctly identify specific dog breeds. It is not surprising, then, that a 2018 survey of nearly 2000 veterinarians found that 89.2% of them disagreed that BSL improves public safety [139]. That emerging consensus should also affect policy, as political science research suggests that lawmakers are at least somewhat responsive to expert opinion [140].

We found several instances, in fact, of politicians in Aurora, Florida, and elsewhere attacking BSL as ineffective in enhancing public safety. Take Gavin Newsom, for example. He was a very strong proponent of breed-specific legislation targeting “pit bulls” when he was mayor of San Francisco in the mid-2000s [141,142]. Thirteen years later, however, he completely changed his position when he successfully ran for governor of California. The website for Newsom’s 2018 gubernatorial campaign informed voters, “He knows that dog breed-specific laws are ineffective at enhancing public safety and jeopardize the welfare of dogs identified as belonging to specific breeds” (quoted in [143]). The Obama White House [58] similarly stated: “*We don’t support breed-specific legislation—research shows that bans on certain types of dogs are largely ineffective and often a waste of public resources.*”

This ineffectiveness frame quite nicely complements our focus on how changing portraits and opinions of “pit bulls” undermined BSL. These two factors, that is, have almost certainly interacted with each other to change policies toward PBTDs. It is much easier for lawmakers to accept scientific research and expert testimony when public opinion is opposed to BSL and “pit bulls” are increasingly portrayed positively than it was when PBTDs were depicted as deviants who posed an immediate threat to public safety. Indeed, policymakers and the courts regularly ignored experts and scientific evidence when enacting and upholding BSL during the “pit bull panic” era (e.g., *Federation of Wisconsin v. City of South Milwaukee,* 178 Wis.2d 353; *Dias v. City and County of Denver*, Civil Action No. 07-cv-00722-WDM-MJW).

Our findings have some noteworthy normative implications, too. Political scientists generally treat responsiveness to public opinion as a positive, especially when it improves policy outcomes. The results in this article are consistent with those criteria. After all, the erosion of BSL in response to changing portraits and opinions of PBTDs moves policy towards the position of most experts in the field and away from prevalent cognitive biases that were driven by the media sensationalism of the late twentieth century. One prominent factor behind BSL, for example, was the misperception of risk from “pit bull” attacks [144]. The panic era’s media coverage contributed to inflated risk assessments by focusing on high-profile incidents that overshadowed the very low overall probability of being attacked by PBTDs (also known as the base rate fallacy). Those portrayals further fueled what social psychologists call “foundational attribution error”—the tendency to attribute bad behavior to dispositional traits (e.g., inherent aggressiveness) instead of situational factors (e.g., poor socialization). In contrast with that flawed reasoning, studies show that external factors (e.g., human behavior) are much stronger predictors of dog bites than breed [4,145,146,147].

While shifting policy away from cognitive biases is normatively positive, we would be remiss in not mentioning a couple of the more problematic implications from our analyses. It is likely, for starters, that the growing popularity of PBTDs shown in this article rests in part on distancing “pit bulls” from their longstanding association with Black culture and owners. Political science research shows that policies, such as drug treatment and governmental spending, are less popular when they are associated with Black people [148,149,150,151]; and the changing portraits of PBTDs discussed throughout the article overwhelmingly have white faces attached to them. Guenther [27], (pp. 184) describes a similar phenomenon among rescue organizations who “*work to make pit bulls whiter by inscribing them with animal practices associated with whiteness, thereby rendering them appropriate and desirable for white homes*.” Pit bull positivity therefore runs the risks of unintentionally reinforcing the same racial stereotypes that helped make PBTDs pariahs in the first place. In addition to those concerns, some animal rights advocates may also find it troubling that the fate of “pit bulls” depends heavily on people’s socially constructed perceptions when humans themselves are largely responsible for the canine behavioral problems that led to regulations like BSL.

The implications of our analyses are much less ambiguous for PBTDs and the people who care about them, though. The social construction of “pit bulls” as deviants who threaten public safety has changed significantly over the past two decades; and in keeping with the scholarly literature on the social construction of target populations, those positive shifts in portraits and opinions of these dogs are currently undermining breed-specific legislation across the U.S. They have already helped overturn hundreds of local laws targeting PBTDs; and positive portraits of “pit bulls” are particularly present in the emergence of a new wave of state legislation aimed at preventing insurance companies from discriminating against owners of PBTDs. Simply put, these changes should significantly improve the welfare of “pit bulls” in the United States.

## 6. Conclusions

We began our discussion of BSL in the introductory section by noting how the Village of Tijeras’s justification for its 1984 “pit bull” ban spread quickly throughout the United States. That model legislation, however, was repealed in 2020 with the village’s mayor enthusiastically proclaiming, “*the bully breed ban is no more. Ring it from the mountaintops*” quoted in [152]. Tijeras, of course, is just one of the hundreds of local governments that have repealed their discriminatory policies against PBTDs in the past decade; and like the Tijeras mayor, many politicians now justify those repeals by evoking the unfair discrimination frame frequently found in positive narratives of “pit bulls.”

To be sure, there is a very long way to go towards ending prejudice and discrimination against PBTDs in the United States and around the globe. These dogs are still portrayed unfavorably by the news media [31], and there are strong proponents of BSL, such as DogsBite.org and Merrit Clifton’s *Animals 24/7*, who counter pit bull positivity with negative stories, public safety frames, and advocacy for victims of dog attacks. A significant minority of the U.S. population, meanwhile, continues to believe that “pit bulls” are unsafe and supports banning these dogs from residential neighborhoods. There are also hundreds of local laws banning and/or restricting ownership of PBTDs that are still on the books in the USA; and efforts by state legislators in Arkansas and Iowa to pass legislation preempting local governments in their states from implementing BSL came up short in 2023. Moreover, these state-level preemption laws frequently do not go far enough to protect these dogs and their owners. We noted that Florida’s 2023 law still allows private entities, such as landlords and insurance companies, to restrict where pit bulls can live; and some localities in states with preemption laws have used ostensibly breed-neutral legislation to disproportionately discriminate against PBTDs. Several countries outside of the U.S. have nationwide bans or breed-specific regulations on PBTDs as well.

But the tide is clearly starting to turn in these dogs’ favor. Pit bull positivity should only continue to grow stronger, too, as each successive generation who came of age with increasingly positive portraits and narratives replaces older Americans whose negative opinions were formed in the pit bull panic era. If so, then we will almost certainly see further erosion of breed-specific legislation and greater anti-discrimination protections extended to owners of PBTDs in the United States going forward.

## Figures and Tables

**Table 1:** (OLS) Time as a Predictor of the Share of U.S. Adults who Like the American Pit Bull Terrier and the Staffordshire Bull Terrier, Q2-2021 to Q4-2024.

	American Pit Bull Terrier	Staffordshire Bull Terrier
Quarter of Survey	0.459 (0.070)	0.414 (0.075)
Constant	50.11 (0.633)	43.96 (0.683)
R^2^	0.767	0.700
Number of Quarters	15	15

Source: YouGov Quarterly Tracker Surveys.

## Data Availability

The full dataset for our team module of the 2018 Cooperative Congressional Election Survey can be found here: https://doi.org/10.1371/journal.pone.0305959.s002. The other data used in the study are all publicly available.
